# Creating safe spaces to prevent unintentional childhood injuries among the Bedouins in southern Israel: A hybrid model comprising positive deviance, community-based participatory research, and entertainment-education

**DOI:** 10.1371/journal.pone.0257696

**Published:** 2021-09-22

**Authors:** Anat Gesser-Edelsburg, Yousif Alamour, Ricky Cohen, Nour Abed Elhadi Shahbari, Rana Hijazi, Daniela Orr, Liat Vered-Chen, Arvind Singhal

**Affiliations:** 1 School of Public Health, University of Haifa, Haifa, Israel; 2 The Health and Risk Communication Research Center, University of Haifa, Haifa, Israel; 3 Beterem Safe Kids Israel, Petah Tikva, Israel; 4 Department of Communication, The University of Texas at El Paso, El Paso, Texas, United States of America; 5 School of Business and Social Sciences, Inland University of Applied Sciences, Hamar, Norway; Drexel University School of Public Health, UNITED STATES

## Abstract

**Background:**

Despite several intervention programs, the Bedouin population living in the Southern District of Israel has the highest mortality rate among children and adolescents from unintentional injuries. Our research questions asked: (1) How does increasing the involvement and participation of Bedouin community members influence the issue of unintentional injuries among children? (2) How does reframing of the technical issue of safety into security influence community involvement and cooperation?

**Objectives:**

1) To identify effective and efficacious positive deviance practices through community-based participatory research with adults, children, and professionals in the Bedouin community. 2) To create wider and deeper connections and cohesion between and among diverse Bedouin communities by seeding and sparking opportunities for social networking and cross-learning.

**Methods:**

The study used a qualitative multi-method approach to generate a hybrid intervention model for reducing unintentional childhood injuries among the Bedouins. To frame the issue of unintentional injuries from the lived perspective of the Bedouins, we employed the Positive Deviance (PD) and Community Based Participatory Research (CBPR) approach. Drawing upon theatrical traditions, entertainment-education (EE), was employed as a way to narratively engage and persuade the Bedouins.

**Results:**

Our research resulted in: (1) the emergence of several PD ideas and practices for preventing and avoiding children’s injuries; (2) the actual creation of a safe and secure playroom for children at a neighborhood mosque; and (3) the creation of cascading and cross-learning social networks between and among members of the Bedouin community spread across various locations.

**Conclusion:**

This study helped in reframing the technical issue of accidents and safety into the notion of sacredness and security, enhanced the association between emotions and cognition by means of experiential and EE methods, and stimulated creative thinking and the emergence of new culturally and contextually relevant ideas and practices through the PD process. It demonstrated the synergistic power of using a hybrid model that combined the rigor and vigor of different health communication approaches to address a significant disparity in the burden of child accidents faced by the Bedouins. Our study generated solutions that emerged from, and directly benefitted, Bedouin children—those, who face overwhelming risk of injury and death from preventable accidents.

## Introduction

Injuries are defined as external or internal harm to the body caused by physical force or by the introduction of a toxic substance [1, 2], and are classified as unintentional or intentional. An unintentional injury is not caused on purpose or with intention to harm, as in the case of motor vehicle collisions, falls, poisoning, drowning, and suffocation. An intentional injury, on the other hand, results from purposeful human action, whether directed at oneself or others.

According to the World Health Organization (WHO), childhood injuries represent a global problem responsible for over 250,000 annual deaths among children age 1 to 9 [3]. While mortality rates from infectious diseases such as influenza and pneumonia have decreased in industrialized countries, unintentional injuries, particularly those due to drownings, falls, and road accidents, are significant contributors to childhood mortality [4, 5]. Indeed, among children ages 1 to 4, injuries are responsible for 12% of the overall mortality rate, while in the 5 to 9 age group, the mortality rates are as high as 25% [3].

According to Beterem Safe Kids Israel, an organization that promotes child safety in Israel [6], in the period from 2014 through 2018, an average of 124 children died each year due to unintentional injuries. For the 2015 to 2017 period, unintentional injuries accounted for an average of 26,000 hospitalizations and 206,000 emergency room referrals annually. According to data from the Ministry of Health, unintentional injury ranked as the third leading cause of death among Israeli children from birth to age 14 [7].

Based on Beterem’s report [8], in Israel, as in other developed countries, unintentional injuries are a function of three interrelated components: child, environment, and parents. The nature of these accidents is shaped by the dynamic interrelationship among these components, including which one is more dominant [9, 10]. In terms of case frequency, the main causes of childhood mortality resulting from home and leisure accidents are drowning, suffocation, falls, poisoning, and fires/burns [11].

An examination of mortality data in Israel from 2016 to 2020 reveals that the chance that an Arab toddler up to four years of age will be killed in an accident are 3.4 times higher than of a Jewish toddler. This disparity increased further in 2020: Among Arab toddlers, there were 12.2 deaths per 100,000, compared to one death per 100,000 among their Jewish counterparts [8].

The target population for this research project included the Arab Bedouin population living in the Southern District of Israel, also known as the Negev. This group has one of the highest mortality rates from unintentional injuries among children and adolescents—a whopping 16.7 cases per 100,000 children. The risk that an Arab child living in the Southern District will die as the result of an unintentional injury is three times higher than that of an Arab child living in any other Israeli district [8].

### Health status in Bedouin society

According to Almassi and Weisblei (2020) [12], at the end of 2019, 268,867 Bedouins—members of various Arab nomadic tribes—resided in the Negev. The Bedouins account for about 20% of the entire population of the Negev and about 14% of the entire Arab population of Israel. About 70% of the Bedouins in the Negev live in 19 recognized localities, while the other 30% belong to 22 tribes living in unrecognized localities—referred to as the Bedouin diaspora [13].

Life expectancy among the Bedouin is lower than in the general Arab population and far lower than the Jewish population in Israel. As per 2008 data, Bedouin men live three years less than men in the general Arab population and seven years less than Jewish men. Bedouin women live four years less than women in the general Arab population and six years less than Jewish women. One of the key indicators of a population’s health status is its infant mortality rate. In 2006, infant mortality among the Bedouin population in the Negev was 11.9 per 1,000 live births—three times higher than the infant mortality rate for the general population in Israel. The main causes of infant mortality in the Bedouin population are congenital malformations and hereditary diseases, attributed in part to the prevalent Bedouin custom of intermarriage between relatives. During the period 2004 to 2006, some 22% of infant deaths in Bedouin society were due to premature birth and its complications [14].

The dilapidated housing and civic infrastructure in Arab localities and the poor socioeconomic conditions of Arab families have exacted a heavy price in children’s lives, including deaths caused by unintentional injury [8]. Based on the latest report published by the Beterem organization [8], child mortality for the period 2016–2020 from unintentional injuries among Bedouin children aged 0–9 years in Israel was caused by (in descending order): trampling (33%), burns (12%), falls (8%), suffocation (3.5%), and other causes (43.5%). A study by Abu-Asba et al. (2009) [15] on child safety knowledge, attitudes, and practices among Arab parents showed that 75% of parents did not install stair railings in their homes, 30% did not install guardrails on balconies, 30% did not fence their roof terraces, and 20% did not take the necessary environmental precautions to prevent childhood poisoning. The study further found that 22% of parents believed that home injuries are a matter of fate and hence not preventable. This belief is especially strong among Arab Bedouin parents with low socioeconomic status.

The effects of poverty among Arab families are exacerbated by large family size, unemployment or underemployment, as well as parents’ indifferent attitudes toward the education of their children [16]. Abu-Saad (2001) [17] emphasized the ill effects of Arab parents being “absent” in their children’s education, and the Goldberg Report (2008) [16] noted the deficiency in Bedouin society of educational support infrastructures such as libraries, clubs, public gardens, and playgrounds. Krupp (2017) [18] pointed out the lack of leisure infrastructure among the Bedouin and the unorganized nature of recreational activities for children aged 3 to 11. According to Ben-Rabbi et al. (2009) [19], very few permanent Bedouin localities have community centers that offer organized activities, but due to economic and cultural reasons only a small proportion of children participate in these activities.

### Intervention programs in Bedouin society to prevent unintentional injuries

Theoretically-speaking, most home accidents and unintentional injuries are preventable [20], and thus prevention programs are directed at educating Bedouin parents in a top-down manner. Moreover, the guidelines are general and addressed to all parents in Israel, without any cultural adaptation. Interventions about safety begins with parents when a child is born, and almost certainly prior to the mother’s release from the hospital. For children who have been injured and hospitalized, parental instruction about preventing accidents begins before their children are discharged [8]. Some accident prevention programs are offered in kindergartens that use play and simulated activities to turn children into active agents of change. In certain youth leadership and student-centered programs, young people are trained in working with parents to create safe home environments [21]. Further, child safety organizations in Israel such as Beterem often organize guidance meetings and accident prevention forums for influential religious leaders [22]. Beterem also runs educational programs in cooperation with Bedouin schools and community centers to prevent home accidents. Through these programs, trainings, and forums organized by Beterem staff members, participants acquire knowhow and tools for creating a safe and accident-free home environment [8].

In this study, we present research findings that suggest concrete ways to reduce unintentional childhood injuries in the Bedouin community. We use a hybrid model that brings together three health communication approaches: (1) positive deviance, (2) community-based participatory research, and (3) entertainment-education. We describe these in the sections that follow.

### Positive deviance approach

The Positive Deviance (PD) approach is based on the premise that in every community there are certain individuals or groups whose uncommon behaviors and strategies enable them to find better solutions to problems than their peers, even when facing more serious challenges and having access to the same resources. PD differs from the top-down expert-driven “best practice” approaches to solving problems. It identifies what a select few are already doing successfully (endogenous practices) and then through a bottom-up process enables the community to amplify those practices [23]. The PD approach [24–26] identifies the behavioral practices of positively deviant individuals within the community and capitalizes on existing community social networks to distribute and implement these practices over time and across locations. Social network maps [27] help in determining the influential pathways through which knowhow and child safety practices can spread through community leaders and other influencers. The four basic steps in the implementation of PD by members of a community [28, 29] include:

Step 1: Define the problem and establish a measurable outcome goal.
Step 2: Identify and determine whether there are certain people or groups—"positive deviants"—who are achieving better outcomes than their peers.
Step 3: Discover the behaviors and strategies that enable the PDs to achieve better outcomes, that is, the PD practices.
Step 4: Design and diffusion: design a process for people to practice the PD behavior, and work in partnership with key stakeholders, including potential adopters, to disseminate these practices through existing and cascading social networks within the community.

According to the Positive Deviance Collaborative [30], PD has been applied effectively over the past three decades in over 65 countries, with a total outreach of more than 30 million individuals. The PD approach has been implemented to address a wide variety of social problems, including reducing childhood malnutrition, enhancing school retention, eliminating neonatal mortality, limiting HIV transmission, improving sales force productivity, fighting against female genital cutting, enhancing health care services, reducing transmission of healthcare associated infections, and enhancing pregnancy outcomes [31].

The PD approach was first applied systematically in the early 1990s to solve the complex problem of child malnutrition in Vietnam. In village after village, program implementers looked for children who came from extremely poor families and yet were well nourished. These children were “positive deviants”—“deviants” as they represent statistical outliers and “positive” because they had solved the problem with no extra resources. For instance, in certain villages the caregivers of well-nourished children fed their children actively, so no food was wasted, and added small shrimps and crabs from their rice fields and sweet potato greens from their gardens to their pho. While these resources were accessible to all, these behaviors were not the norm. Once identified, these PD behaviors were practiced by caregivers in group cooking and feeding sessions, and rates of malnutrition dropped between 65% and 86% in villages where the program was implemented [32]. Inspired by the success story in Vietnam, the PD approach has been applied widely to address various child protection and welfare issues: combating malnutrition, reducing maternal and infant mortality, tackling sex trafficking among children, and reintegrating child soldiers [33]. To the best of our knowledge, no PD project has dealt with preventing childhood accidents and creating safe play environments. Certainly, none has been implemented with the collaboration of the children themselves.

### Community-based participatory research

We also employed community-based participatory research (CBPR) in our project as a parallel and complementary approach to positive deviance. Rather than the traditional expert-driven approach [34], CBPR is a bottom-up research approach that engages community members not as subjects but as partners. The community is involved in every stage of the research, including in identifying their salient problems. CBPR is increasingly used to examine health inequities and promote health equity in communities. Brush et al. (2020) [35] emphasized that effective CBPR partnerships require time to build the ground infrastructure, institutional and interpersonal relationships, and vital capacities to realize partnership goals. Ongoing processes require monitoring across partners, partnerships, and outcome dimensions.

In the past decade, more and more CBPR studies have focused on child health issues, offering a unique approach to translate evidence-based models and research knowledge about childhood health into effective and sustainable interventions [36].

### Entertainment-education approach

Another approach utilized in this project is the entertainment-education (EE) approach. In this approach, educational content is deliberately integrated into an entertainment program with the aim of boosting knowledge about a particular subject among a target population, creating or strengthening positive (or negative) attitudes toward this subject, and changing behavior accordingly [37]. A wide variety of media platforms can be used to promote EE, among them theater, television, cinema, computer games, comics, and social media.

Entertainment and drama platforms are particularly useful in addressing sensitive issues (such as childhood accidents) by creating a rich and nuanced narrative that goes beyond simple slogans. Mass-mediated EE messages can reach target populations beyond the ones trained in face-to-face child safety programs. Exposure to drama or entertainment programs on risk prevention of child accidents, for instance, can facilitate experiential learning, boost emotional involvement, and trigger desirable action.

Based on various theories and models of behavior change and influence, the EE approach holds great potential in general, and for prevention of childhood injuries in particular [38, 39].

Art-based scholarship, whether it involves music, drama, literature or the visual arts, values the centrality of the human experience vis-à-vis works of art. In this sense, art can serve as a creative research tool that allows for in-depth investigation of human-centered trauma and therapy [40].

Drama and entertainment can animate social issues in an engaging and non-didactic way and tackle complex issues through a multiplicity of characters and perspectives. Those watching the drama often lower their defenses and may be transported to a narrative space where they can be influenced to change their perceptions, attitudes, and health behaviors. Drama can help audience members sort through dilemmas about how to behave in risk situations through spurring of conversations into the private, community, and public spheres.

Research on EE lends itself to pluralistic research methodologies combining quantitative and qualitative tools—e.g., audience surveys, in-depth interviews, focus groups, participatory photography, sketching, and art. The goal in art-based research is to use the arts as a method and form of analysis within a qualitative research framework [41]. Through images, narratives, poetry, and dramatic plays, researchers can grasp the meaning of a person’s experience [42, 43].

Research findings show that purposely and systematically implemented EE together with formative research, on-the-ground partnerships, and service delivery can lead to increased involvement of audience members as well as engender significant changes in their knowledge, attitudes, and behaviors with respected to an issue [37, 44, 45]. Such effects occur as a result of the high degree of exposure received by EE programs and their ability, over time, to raise issues in a deeper and more complex way than a single-shot advertising campaign.

Given the importance of preventing unintentional childhood injuries and the serious dearth of field-based projects that are strategically anchored in health communication approaches (e.g., PD, CBPR, and EE), we devised a hybrid model to guide our study.

Given the high rates of unintentional injuries among Bedouin children despite the existence of various intervention programs, our research questions asked: (1) How does increasing the involvement and participation of Bedouin community members influence the issue of unintentional injuries among children? (2) How does reframing of the technical issue of safety into security influence community involvement and cooperation?

### Study objectives

Our study objectives were four-fold:

1. To identify new positive deviance practices through community-based participatory research drawing upon the existing wisdom of adults, children, and professionals in the Bedouin community.
2. To create connections and cohesion between and among various Bedouin communities through social networking and cross-learning opportunities.

## Materials and methods

### Research design

**Fig 1:**
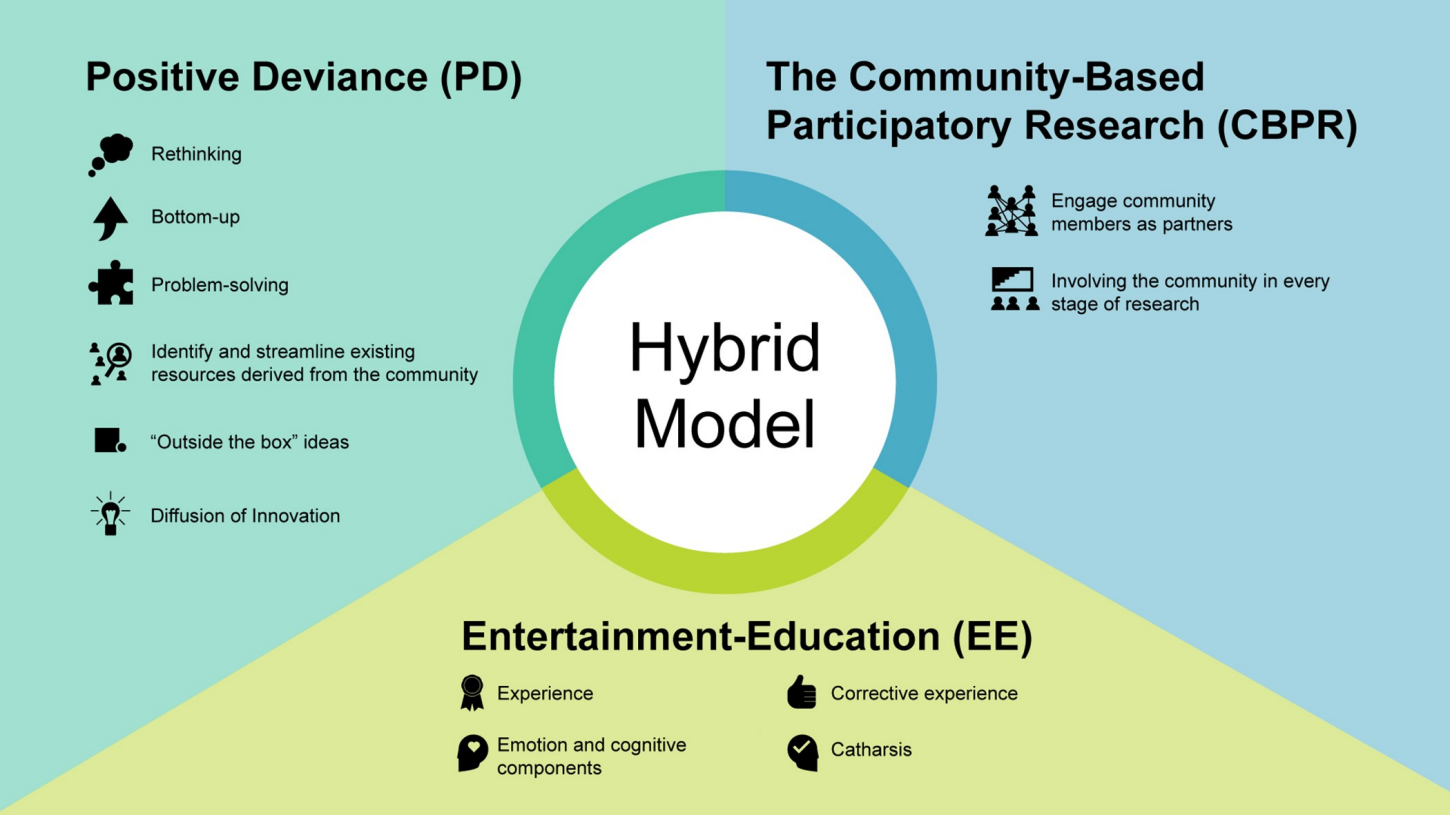
Hybrid model: Reframing the issue of child injuries and the motivation for action. The hybrid model incorporates a combination of three approaches: Positive Deviance (PD), Community-Based Participatory Research (CBPR), and Entertainment-Education (EE).

Our research consisted of multiple qualitative methods while drawing upon the three health communication traditions—Positive Deviance (PD) [23] and Community-Based Participatory Research (CBPR), in which issues, ideas, and interventions are derived from the lived perspectives of the target population, and Entertainment-Education (EE) [37], which uses theater as a platform to narratively engage and persuade the target populations. Fig 1 depicts the key conceptual components of the three approaches.

We chose the CBPR approach because it is tailored to minority populations and especially for culturally sensitive issues such as unintentional child injuries. As noted previously, almost intervention programs on child safety are top down—trainings, tutorials, and lectures implemented in schools and kindergartens rather than emerging from the community (from the bottom up). The second component in the model is the PD approach that is akin to CBPR in its bottom-up orientation while purposely identifying "outliers" who have found solutions within their environment without additional resources. The third component is EE—another intervention approach for social change akin to CBPR and PD that purposely employs engaging experiential art-based strategies.

### Research population

The total study population comprised 404 participants from the Bedouin localities of Hura, Segev Shalom, and Lakiya in the Negev. These localities were selected as they represent the population diversity of the Bedouin society. The 404 participants included 101 (25.0%) children aged 3 years to 10 years, 280 (69.3%) women, and 23 (5.7%) men. Table 1 describes the socio-demographic characteristics of the adult participants in the study. Among these participants, we (1) conducted 23 personal interviews with professionals in the fields of safety, education, and welfare working in the Bedouin community, including welfare workers, community workers, teachers and educators, and religious leaders—clerics and imams; (2) conducted 17 art-based focus group discussions with mothers and grandmothers from the Bedouin community in Segev Shalom and Hura localities; (3) conducted five art-based focus group discussions with teachers (including kindergarten teachers); and (4) carried out four-art based focus group discussions with preschoolers and elementary school pupils. In the subsequent design and diffusion phase, interventional ideas and practices were shared in the locality of Segev Shalom and Hura, leading to the actual implementation of a child safety project—the building of a playroom—with 53 participants in Lakiya.

**Table 1:** Socio-demographic characteristics of the adult participants in the study (n = 303).

	Not Employed	Employed		Total
	n (%)	Various areas of employment	Education system employees	n (%)
		n (%)	n (%)	
**Number of participants**	143 (47.2)	87 (28.7)	73 (24.1)	303 (100)
**Average number of children in the family**	5.3	4.8	3.1	4.6
**Average age (years)**	35.3	34.9	32.1	34.3
**Married**	117 (38.6)	82 (27.1)	66 (21.8)	265 (87.5)
**Not Married**	26 (8.6)	5 (1.6)	7 (2.3)	38 (12.5)

### Research process

**Fig 2:**
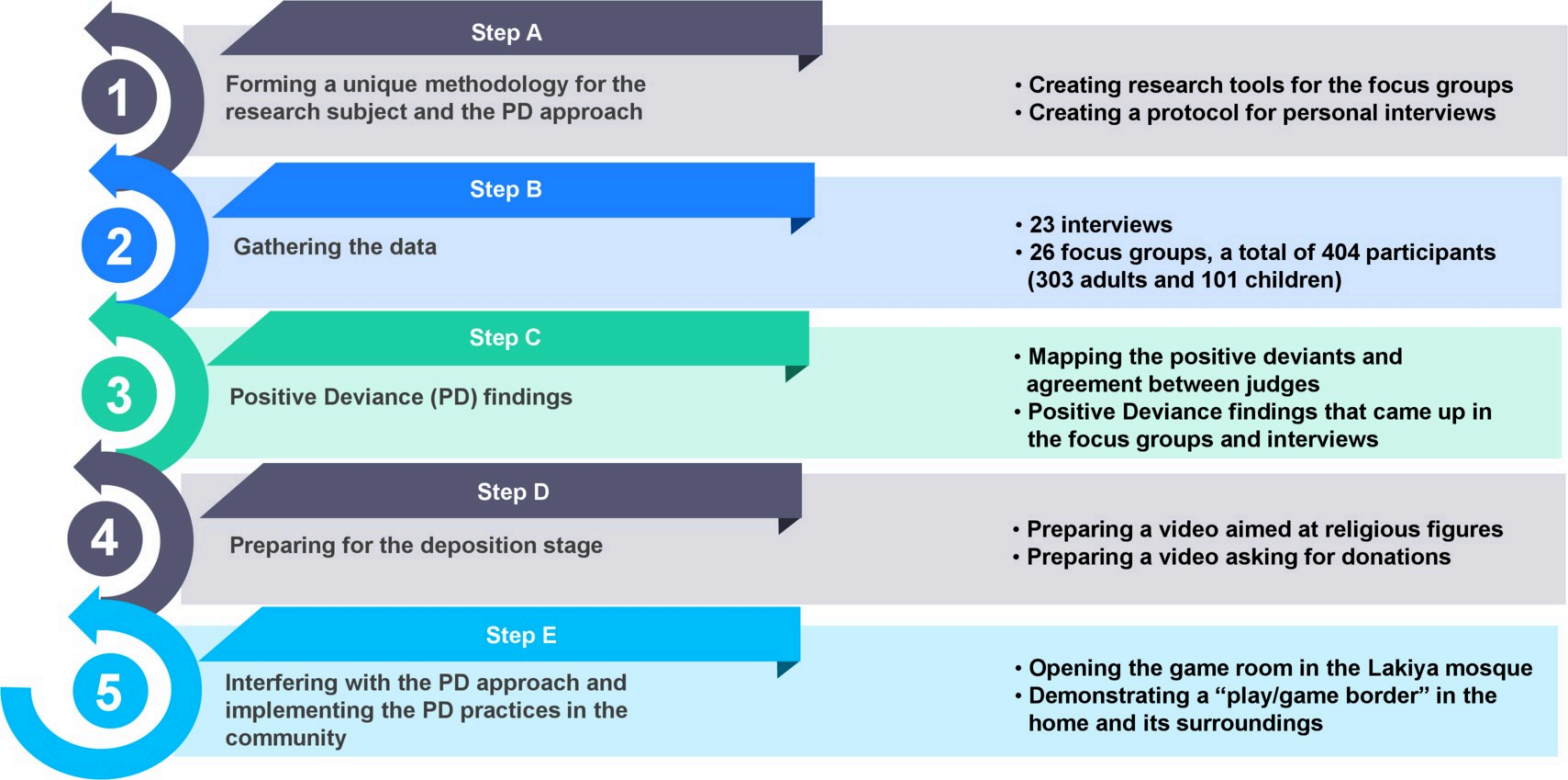
General structure of the research process. The research process incorporated five steps: (A) devising a unique methodology for the research topic and the PD approach; (B) gathering the data; (C) identifying the PD findings; (D) preparing for the design stage; and (E) diffusion of the PD practices in the community.

The study was carried out during an eight-month period from June 2019 through February 2020. Consistent with the PD approach, we wished to identify households in which no child accident was reported over the past five years. As per the PD approach, these households would have held insights, ideas, and practices that protected children from unintentional harm. However, consultations with the Beterem organization and with hospitals in the southern district of Israel made it clear that such information was not easily available for reasons of medical confidentiality and patient privacy. Hence, we had to adapt our approach to the local conditions and began conducting interviews and art-based focus groups with community members from the Hura and Segev Shalom localities (see Fig 2).

A snowballing approach was used to reach as many people as possible to increase the probability of finding positive deviant households among the Arab Bedouin families. Almost instantaneously, the data collection yielded unusual ideas and practices for prevention of unintended accidents not found in official safety guidelines. After the data collection phase, we mapped these unusual and uncommon practices and validated them through sessions with safety experts, who assessed their efficacy relative to the official safety guidelines. To be included, each validated practice satisfied two conditions: (1) The practice is unique and not included in official community guidelines and safety rules. (2) The practice can be applied immediately within the existing social, cultural, and economic resource base of Bedouin society. Using an iterative and converging process, investigators had to reach complete agreement on a unique practice for it to be deemed a PD practice. The diffusion phase included a video-based strategy to disseminate the PD practices that were identified and to validate them among the larger Bedouin population. The diffusion phase also included purposive assimilation and incorporation of innovative ideas proposed by children regarding the creation and construction of safe and secure spaces—for instance, the creation of a playroom in the local mosque (as detailed later).

### Research tool: Using entertainment-education in an art-based focus group to reconstruct a story about childhood injury

Because many mothers in the Bedouin population have very low health literacy and may be normatively constrained to take independent decisions and actions [46, 47], we crafted an appropriate research tool to engage them. The tool entailed the use of an EE-based platform consisting of theater and dialogue games to enable the participants to express themselves fully in the art-based focus group discussions.

Among the different art-based possibilities, we chose to focus on a theater-based approach [48]. We video recorded all the theatrical encounters in order to collect all insights and materials that arose in this dynamic dialogic space. These included dramatic situations, dramatic roles, on-stage narratives, use of language and metaphors, expansion of characters and roles, movement in space, aesthetic patterns, and more. The multidimensional processes that occur dynamically in a dramatic space facilitate observation of the narrative from various angles, thereby creating room for interpreting and analyzing the emerging materials [49]. That is, the narrative emerging on the dramatic stage by itself constitutes a unit of research analysis.

The protocol for the community art-based focus group session (S1 Appendix) was crafted in line with the theatrical practices of Augusto Boal [50], who pioneered the use of theater for social change. Boal coined the term “spect-actor” to refer to spectators from within the community who become active agents in the dramatic space, i.e., actors [51]. The first stage of the meeting protocol included theatrical warm-up and getting-to-know-you games. In the second stage, which constituted the heart of the meeting, the facilitator told some stories—in a deeply compassionate tone—about unintentional child accidents involving burns, trampling, and suffocation. These narratives were based on actual events within the community and hence were authentic and able to capture the participants’ attention. The participants were then invited to act out the story told by the facilitator. In doing so, the participants were told to represent the characters and interact with the unsafe environment—for instance, negotiate stairs without a guardrail, intervene with a car preparing to back up a driveway.

The third stage entailed implementing the joker exercise from Boal’s theater [50]. The joker is a neutral facilitator who encourages the “spect-actor” to solve the problem of unintentional and accidental childhood injuries. The joker asked the audience members to change whatever needed to be changed to solve the problem. Participants were charged with proposing specific ideas and actions to prevent the child from being injured. They made suggestions for change and acted out the story while modeling their suggestions in detail. As the desired goal was to prevent the child from being injured, the proposed changes actually led to deconstructing and reconstructing the injury narrative.

In the final phase of the session, the participants watched a videoclip called "Lynn’s Story" in which a young Bedouin girl named Lynn asks the community to think of ways to keep children safe and to make suggestions for creating safe and feasible play environments. “Lynn’s Story” was inspired by a UNICEF project in Colombia in which a young fictional Colombian girl, Juanita [52], sends a video to all mayoral candidates asking them to create a safe and healthy environment for children. In making this request, she holds them publicly accountable for childhood safety. Similarly, “Lynn’s Story” served as a means for Bedouin children to ask and inspire the community adults to take action to keep them safe.

After watching “Lynn’s Story,” focus group participants discussed ideas and practices that could be implemented locally to improve child safety. At the end of the art-based focus group, participants were provided with a magnet—a token of their participation. On the magnet were two sentences that served as affirmations and action cues: (1) A verse from the Qur’an expressing the importance of children in Bedouin society: "Money and children are the decoration of life,” and (2) a PD-inspired sentence focusing on action: "What did you do today to provide a safe and secure play environment for me?”

### What constitutes a safe and secure play area for children? Children’s drawings

The art-based focus groups with the Bedouin mothers often brought children into the conversational space. Hence, in the current research we decided, impromptu, to involve the children. We brought toys, arts-and-craft materials, play dough, and other art supplies and asked the children to create scenarios depicting what they considered to be a safe and secure play environment. We also asked them to draw pictures of a safe and secure play environment for themselves located within their localities.

### DAD—Discovery and action dialogue for preventing injuries

The interviews with safety professionals were based on discovery and action dialogues (DADs). While the DAD interview protocol (S2 Appendix) was specifically developed to identify PD practices to reduce hospital-acquired infections, it is equally valid for investigating solutions for other complex problems [24, 53]. DADs make it easy for a group or a community to discover practices and behaviors that enable some individuals from within the community to find better solutions to common problems than their peers. Given that the present study was designed to develop tools with genuine potential to help the Bedouin population reduce childhood injuries, we adapted the DADs for this purpose.

The interview protocol thus focused on: (1) examining attitudes regarding child safety, injuries, norms, risk perceptions, and intervention programs in Bedouin society; (2) identifying social barriers to injury prevention and analyzing cases in which accidents were prevented; (3) identifying positive deviants who had found effective ways to implement child safety despite facing odds; (4) locating PD practices and behaviors within the community, and showing "Lynn’s Story" and inviting people to suggest solutions; and (5) identifying preferred and credible information channels within the community and distilling effective means of conveying information (narratives, visual representations, and questions and answers).

### Reflective discussion: Personal experiences with art based focus group among mothers and grandmothers

Most of the mothers and grandmothers in our art-based focus groups told us that their experience with this research project was different from previous research studies in which they participated. They felt engaged all the time. The adults who participated in the 22 art-based focus groups that used theater games to dramatize accident stories noted strong familiarity and resonance with the subject matter: “We often encounter shocking cases like these.”

During the art-based focus groups, eight of the participants told the others extremely personal stories about unintentional accidents. One participant told the story of her friend who ran over her own daughter. “Before it happened, it never crossed her mind there was any chance her daughter was behind the car.”

Another participant who watched the injury story dramatization commented: “This story reminds me of a similar incident that took place right before my eyes almost ten years ago. It was something that is impossible to talk about and impossible to forget.” She went on: “At 6:00 a.m. I was standing by a window on the second floor of my home. My uncle was on his way to work and he had a two-year-old son. He had a large transit vehicle, not a small car, and he put it into reverse. I didn’t see the boy. I saw a pregnant woman in her seventh month, screaming, ‘The boy… the boy is under the car’ … My uncle did not hear her because the car window was closed. He reversed three times and ran over the boy three times by the time the pregnant woman reached him. The boy was already dead. I saw everything from upstairs and I was not able to do anything. I saw the woman screaming and I could not do a thing….”

Clearly, the stories these participants told were authentic and gut-wrenching.

### Analysis

A five-member research team analyzed the data using thematic analysis. The team examined the relevant themes and sub-themes emerging from the art-based focus groups, the children’s drawings, and the interviews with professionals. Novel ideas and practices for preventing childhood injuries identified, especially those not included in the safety guidelines. To ensure reliability, the five members of the research team each analyzed the data independently. Each researcher read the discussion transcripts to gauge the range of general and potential meanings. Then, each devised an initial coding scheme by categorizing units of text as themes and labeling these units with an appropriate descriptive phrase. In the next stage, the researchers met and created a consolidated rubric of independently identified themes. Next, the first author discussed the themes with the entire team to ensure they were coherent, consistent, and distinctive. These processes led to the generation of a detailed thematic framework for the subsequent analysis. After that, the researchers met with representatives of the Beterem organization to further evaluate and hone the thematic categories. The researchers then convened one more time to make the final adjustments to the thematic map.

### Credibility

Observing complex social phenomena using diverse research tools allows for a broader and richer understanding [54, 55]. In the present study, data collection and processing occurred simultaneously, yielding an integration of perspectives and a comprehensive framework for understand the phenomenon of unintentional injury [56]. We used multiple triangular arrays based on the integration of a variety of research tools addressing the same phenomenon under study (e.g., art-based focus groups, theater plays, personal interviews, and children’s drawings). These arrays greatly increased the credibility [57] and reliability of the study and the interpretation of its findings [58].

All the interviews and art-based focus group sessions were recorded, transcribed, and logged in a comprehensive field diary. This diary enabled the researchers to examine the reliability of the data at each stage, cross check the data, and then confidently proceed with the analysis. The field diary included notes on the time and place of the art-based focus groups and interviews, the dynamics during the meetings, the interviewees’ observed resistance to questions, and notations of nonverbal responses (e.g., body gestures, facial expressions, and tone) that are difficult to infer from typed transcripts. Given the sensitive nature of the topic of childhood injuries, the researchers’ detailed documentation and reflections yielded a more accurate, holistic, and deeper understanding of the data. The art-based focus group protocols were initially written in Hebrew and translated into Arabic, the mother tongue of the research population, and then back translated from Arabic into Hebrew to ensure convergence in meaning and intent (S1 Appendix). In addition, the transcripts of the art-based focus groups were transcribed into Arabic by three researchers who are fluent in both Arabic and Hebrew.

A pilot study with four mothers to test the interview protocols. Moreover, the study participants represented different subpopulations (different age groups—for instance, grandmothers, mothers and children), thus strengthening the credibility and validity of the findings with respect to the studied phenomenon [59].

### Ethics

The study was approved by the Committee on Health and Welfare Sciences, The Faculty of Social Welfare and Health Sciences at the University of Haifa, approval number 189/19. All the study participants gave their written consent to participate in the research and publish its results.

## Results

Our research findings are organized under two headings: (1) Positive deviance ideas and practices for preventing and avoiding children’s injuries; and (2) Spurring and cascading a social network among members of the Bedouin community for rapid diffusion.

### Positive deviance ideas and practices for preventing and avoiding children’s injuries

The themes shown in Tables 2 and 3 represent the integration of the positive deviance ideas and practices proposed by the professionals and the community participants. The ideas are presented in ways that are generally applicable to all types of childhood injuries (falls, burns, suffocation), with some ideas geared specifically to preventing children from being run over. As noted previously, these ideas emerged from the community participants, were vetted by professionals, and did not appear in any official guidelines issued by safety organizations. Table 2 outlines PD practices for generating reminders for parents as well as practices that entail social involvement of community members. Table 3 lists PD practices for setting visual boundaries, practices to avoid running children over, and practices to remind parents not to leave children in vehicles.

**Table 2:** PD practices suggested by mothers and grandmothers for generating reminders for parents and involving community members.

PD practices for generating reminders for parents and involving community members	PD practices that deal with community social involvement
Hang a piece of paper and/or pictures on the front door of the house with a checklist of things to review before leaving the house.	Set up an organized duty rotation among mothers that assigns one mother per day to watch over the safety of the neighborhood children as they play outside (e.g., in the parking lot) in the afternoon.
	“If my house is on the same 1,000 meter lot with three or four other buildings, where is there space for the children to play? Only in the parking lot! Because there is no space to play, how can you keep your children cooped up in the house? So they go out to play on their own. That gave me the idea of finding some safe place in the neighborhood where they can play and setting up a rotation among the mothers so that each time a different mother watches them.” (AN)
Put a daily reminder in parents’ mobile devices to remind them of important things to check before leaving the house.	Create a regular schedule of meetings in which daughters and daughters-in-law, mothers and mothers-in-law, and grandmothers sit together and discuss how to keep children safe. The objective is for the younger generation to hear stories and learn from the past safety experiences of the older generation.
	“For example, if the preschool teachers were to provide instructions for mothers or for groups of women, the women could pass on these instructions to their daughters and neighbors. I think society as a whole can help itself and make a difference.” (HG)
	Take advantage of existing play settings such as neighborhood schools, where facilities exist for the children to play freely and securely in the afternoons.
Teach the children about safety for themselves and their friends through play. “Write notes with them” about what is permissible and what is not, and post these notes on the refrigerator. Each child earns points and gets a small prize at the end of the week.	Incorporate religious principles into safety instructions when provided by religious clerics.
	“Not everyone goes to school. Not everyone goes to the health clinic, the government council, the community center. So I thought about [teaching] those who go to the mosques. For the past six years, I’ve been running a forum with imams and…. I asked them to take part in this effort. I run the forum once in two months. The participants get together for a lecture, a meal and a discussion of a topic I bring up. I’ve also run sessions at hospitals. For example, there was a case of a woman who had seven Caesarian sections and refused to have her tubes tied because it is wasteful and forbidden in Islam. I brought the imams from the mosques to meet with the surgeons at Soroka Hospital, who explained that this woman’s life was in danger. Today, these doctors give women the telephone numbers of these imams. I made the knowledge [about child safety] accessible to the imams, and this has had an impact. This is an example of an initiative that meets the needs of the community.” (AA)
Devise a pre-agreement or a game that the children know they can use when their mother is busy and cannot watch over their play. During this time the children know not to go out to the yard or anywhere dangerous.	

**Table 3:** PD practices by suggested mothers and grandmothers that entail setting visual boundaries to prevent children from being run over or forgotten in cars.

PD practices entailing visual boundaries	PD practices to prevent children from being run over or forgotten in cars
Set visual boundaries for children, for example by painting an area in the yard blue to designate the area where they are permitted to play and painting areas where play is prohibited in red. Use grass or cloth to designate a defined play area.	Place a barrier in the play area outside the house to make drivers slow down and not endanger children’s safety.
“For example, I installed a strip of synthetic grass in the yard. That is where the children can sit and play. This is a designated area and even the neighbors come to play there on Saturdays.” (PV)	
Define a play area somewhere in the house. The area can be delimited using objects found in the home, such as sheets or pillows.	Walk around the car before getting into it and turning on the engine (see video clip).
	“Every day I walk around the car before I start driving. Maybe I have a flat tire? Maybe there is a cat behind the car? Maybe there is a person? This can become a conscious habit for people.” (AA)
Hammer a few nails into the wall and hang a piece of cloth from the ceiling to the floor, like a theater curtain. Every time the mother asks her children to play, this defined space sets the play boundaries.	When you get into the car, honk the horn and wait a minute or more before starting the car.
	When the mother knows the father is about to leave the house, she gathers all the children around her and waits by the door with them until the father or one of the adults drives off.
	When you get into the car, give your mobile phone to your child. When you leave the car, you automatically will look for your phone and not forget to take your child with you.

### Creating a safe and secure play space: Children suggest setting up a playroom at the mosque

When the children were asked to draw a safe and secure play environment, the children from Segev Shalom and Lakiya drew playrooms in the mosque. Mosques in Israel usually do not have playrooms for children; the mosque is customarily used only for men’s prayers or for special events. The children’s original and innovative idea led to a decision to attempt to set up a safe and secure playroom for children in the mosque. The process of setting up the room yielded a number of byproducts, as described below.

#### The first byproduct: A videoclip of children directly broaching the idea with the imams

In this videoclip, the children briefly describe their problem of no safe place to play and ask the imams if they can help by allocating space for a play area in the mosque where they can feel safe and secure.

The videoclip was edited with the cooperation and consent of community members and distributed by the researchers on the WhatsApp groups of the imams and of the community members, as well as on the website of Positive Deviance Israel. Within a month, the videoclip received 45,000 views and 469 shares.

#### The second byproduct: Enlisting community members to set up a playroom in Lakiya

Although a number of imams expressed interest, a decision was made to establish a playroom in the large mosque in Lakiya. The imam in Lakiya allocated an unused room for this purpose and urged the community members to waste no time in turning it into a safe and secure play area.

A number of steps were taken to inform the community about the playroom project and to enlist their help in establishing it.

1. A meeting with the Braya Community of Bedouin Painters from Ben Gurion University of the Negev. They were immediately enthused by the idea and volunteered to help create wall paintings together with mothers from the community.
2. A meeting between the social involvement coordinator at the Lakiya high school and several students. About 54 girls from 10^th^ through 12^th^ grades volunteered to spend two hours per week watching over the children in the playroom.
3. A meeting with the imam, who introduced the researchers to a teacher at the high school who would coordinate the volunteer program in the playroom.
4. A teacher in the field of preschool education gave a workshop for the girls who volunteered for the program. The workshop trained the volunteers by focusing on the attributes of children in this age group and ways of dealing with them in an age-appropriate manner.
5. The volunteers launched a campaign to collect board games and toys from the community. At the same time, a fund-raising campaign was launched to purchase games, art-and-craft materials, and furniture to renovate the room.

### The third byproduct: Preparing the playroom at the Lakiya mosque with the help of the community

1. During the first two weeks of February 2020, twelve students from the Abu Aish neighborhood in Lakiya, where the mosque is located, cleaned the room thoroughly, painted all the walls, and prepared wall paintings suitable for a children’s playroom.
2. Men from the community installed synthetic grass in the playroom.
3. Volunteer mothers collected children’s games from Lakiya residents.
4. Fathers from the neighborhood volunteered their time to clean and fix up the yard and remove any objects or materials that were potentially hazardous.
5. Financial donations by community members were used to buy games from a large toy store in Tayibe.
6. The 56 girls who volunteered to take part in the initiative set up a WhatsApp group. They decided that the playroom would be open every day for two hours and that five volunteers would be present each day and would open and close the room.
7. People from the community prepared a film inviting the children to the playroom opening. The film was disseminated via social networks and social media.

#### The fourth byproduct: Opening the playroom

About 40 children from neighborhoods in Lakiya came to the opening ceremony of the playroom. Volunteers, imams and people from the community noted the importance of a safe and secure playroom for the children. The imam also stated that he, together with community members, would continue to watch over the preservation of a safe and secure environment, removing hazards as they came.

### Creating a social network among members of the Bedouin community

**Fig 3:**
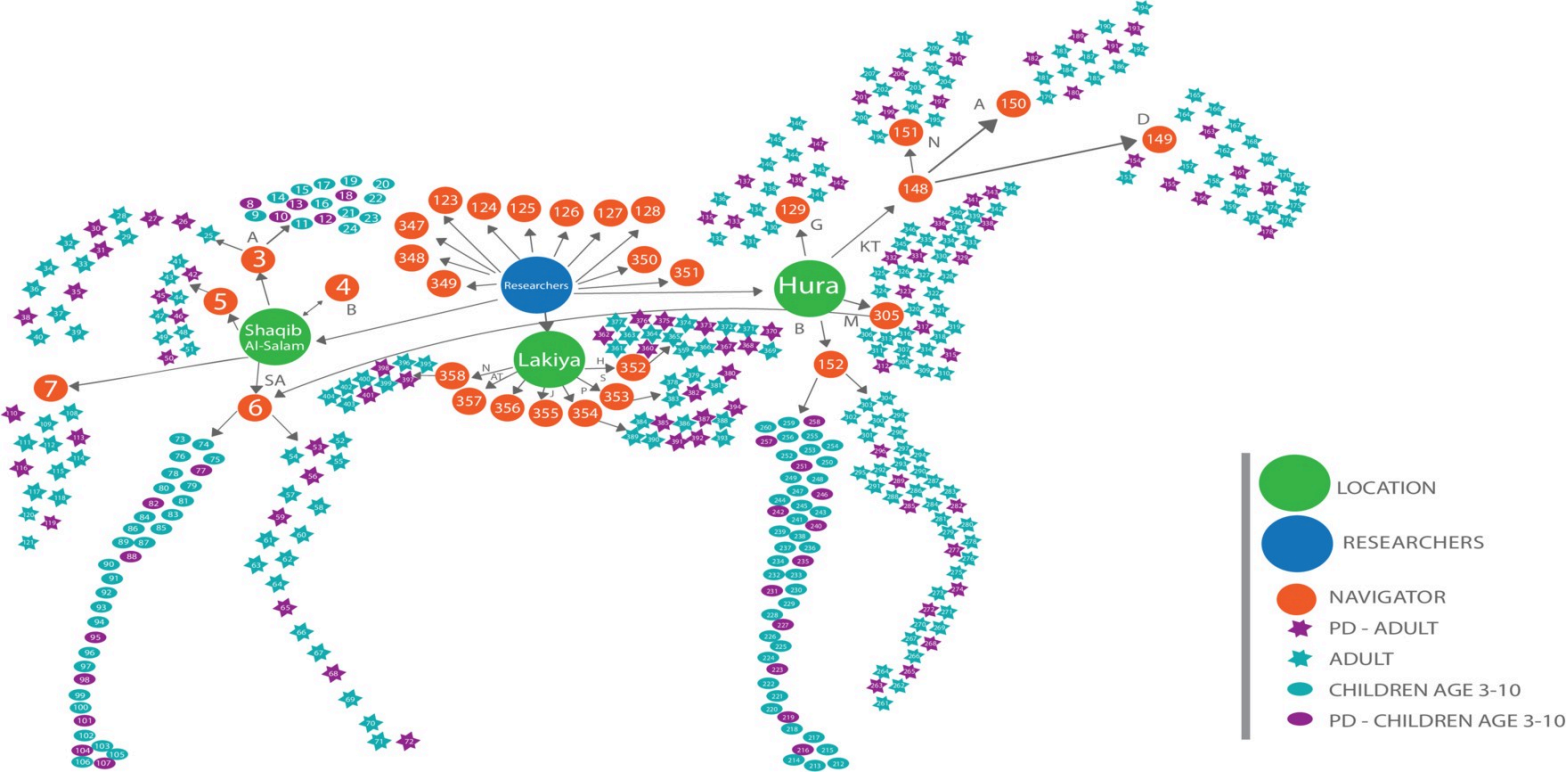
Toy horse depicting new social network. The social network is purposely mapped in the form of a toy horse. Metaphorically, the skin and tendons of the horse stretch across the three Bedouin communities that participated in this study.

Fig 3 depicts the social network mapping the connections between community members and professionals in the three Bedouin localities (Hura, Segev Shalom and Lakiya) where the research was carried out. The color-coded key describes the location and attributes of the 404 participants who took part in the research.

This social network reflects three key insights: First, the network depicts how new ideas emerged from the field through community residents comprising both adults and children. Second, in each community we found people who provided new ideas and key people who opened doors (navigators) so everyone in the community could be reached and invited to contribute. Third, the social network depicts how ideas travel within each community as well as across communities.

Why is the social network represented in the shape of a toy horse? While there is no compelling reason, we chose this symbolic representation based on the metaphor of the Trojan horse in Homer’s *Odyssey*, in which the horse was used as a trick by outsiders to enter and conquer Troy. In contrast, this symbolic horse is not a Trojan horse but rather represents how a new reality can be created from within the existing networks of the three communities.

## Discussion

The findings and outcomes of the present project are a powerful reminder that the wisdom for preventing unintentional childhood injuries resides with the community itself. The practices proposed by the participants for promoting childhood safety included getting community members socially involved, suggesting reminders to parents using checklists and cell phones, establishing visual boundaries for play, and providing tips to prevent children from being run over and being forgotten in cars.

It is important to note that the new ideas and practices suggested by the community were not found in the official instructions or guidelines issued by the hospitals or the Beterem organization in Israel.

The PD practices we found have heuristic value in that most of them can be applied to all kinds of injuries: burns, suffocation, falls, being run over by cars. Not only do mothers and grandmothers have tools to create a safe and secure play environment for children, it also reminds children of the boundaries that will protect them. In addition, the proposed ideas included specific practices to prevent children from being run over—the most common child injury. For example, before a father begins driving the car, he automatically walks around the car to make sure no one is hiding there.

Further, it was the children’s drawings that raised the idea of setting up a safe and secure play area in the mosque, leading to the establishment of a playroom in the Lakiya mosque. Note that prior to the present research project, there was no such playroom in any of the mosques in the Bedouin localities.

In the following discussion, we examine the possible reasons that this project was effective both in proposing new ideas and practices for preventing childhood injuries and, more importantly, in implementing them. Our interpretation is guided the components of the study’s hybrid model, which is based on EE (entertainment-education), CBPR (community-based participatory research), and PD (positive deviance).

The entertainment-education [60] approach enabled the participants to express themselves in novel ways. By means of Augusto Boal’s theater games [50, 51], the participants were able to dramatize their injury stories and then as “spect-actors” suggest how to prevent such stories from recurring. This new possibility of experiencing injuries through a dramatic narrative was a unique means of fostering involvement and generating ideas, which differed from what they had experienced previously. The Bedouin community was familiar with top-down intervention methods (lectures and trainings) and content to serve as passive interview respondents for externally conducted research studies [8, 21]. When such methods are employed, participants tend to provide cognitive responses. In contrast, the dramatic research protocols used in this study roused their emotions and elicited new ideas and actions. That is, by means of the injury story narratives, the participants actually put themselves into the stories and fully experienced them both emotionally and cognitively. Not only did they “talk about” the injury, but rather through the theater games they actually helped write the injury story. At the end of the dramatization, the participants were instructed to think about how the injury could have been avoided, thus providing them a generative space to tap into their collective problem-solving wisdom.

The use of theater/drama enabled the participants to be drawn into the process at a deeply personal level. They were able to let down their defenses. By either participating in the dramatizations themselves or watching their friends, they experienced the situation vicariously i.e., through an indirect mechanism. Also, most participants were familiar with stories of childhood injury in their immediate environment. Research suggests that the opportunity to engage with the injury story with others likely helped ease their personal pain and feelings of guilt. Moreover, dramatizing the injury story to the point of preventing it may have created what is often termed a “corrective experience”—the ability to experience a correction by changing the narrative of how to prevent the child from being injured.

Processing the issue of children’s accidents by means of entertainment-education likely enabled the participants to undergo a process of catharsis. In his *Poetics*, Aristotle coined the term “catharsis,” simply translated as “an intellectual clarification of [the] events” [61]. For meaningful change to occur, an audience member must arrive at a new understanding, i.e., must experience a moment of catharsis. During the theater games, the participants likely experienced both pleasure and meaningful learning. They underwent a process of engaged learning in which new interpretations, positions and possibilities emerged. As Gesser-Edelsburg and Singhal (2013) assert [38], these new positions are modeled and reinforced through various characters in other EE events. In the process, spectators feel increasingly sure of their potential to carry through on new positions and implement new insights. In the present study, the new insights and ideas were in the domain of injury prevention.

Moreover, in this study, the issue of injury was reframed in a new way. Note that in English the word “safety” incorporates both the official aspect of being physically safe and the emotional aspect of feeling secure. The art-based focus group discussions in this study were conducted in Arabic. Arabic uses one word (أمن) to express the notion of conforming to professional safety regulations and another word to express the emotional aspect of feeling secure (مكان آمن). We asked the community to propose ideas and practices that would help in creating a secure (مكان آمن) play environment. We asked the children to describe a play framework in which they would feel secure. In these questions, the transition from using the official term referring to safety regulations to the term representing the concept of emotional security is not merely semantic, but rather intentional and fundamental. We believe that the use of the Arabic word for security encompasses a component that arouses emotions and identification, as opposed to using the general notion of safety, which represents a more objective and official concept. The notion of a sense of security encompasses a subjective component for each of the participants, so that the listeners ask themselves the following question: “Where do I feel most secure and protected?” (i.e., for children) and “How can I create a secure and protected environment for my children?” (i.e., for mothers/grandmothers). In other words, the participants do not merely ask themselves about the rules for safety, but rather about the conditions for creating a protected and secure environment. We believe that the use of the Arabic word for security (أمان) motivated the participants to think about the topic of injury from their own personal perspective, allowing them to delve into their inner worlds.

In this study, the target audience also included children—whose safety and well-being were drivers of the study. This is important for a number of reasons. First, we treated the children as partners. Usually the matter of safety is considered to be the exclusive responsibility of adults. By inviting and engaging children to think about a safe and secure environment, and using their voices as a means of promoting community action, we incorporated the fundamental principles of community-based participatory research [34]: inclusion of the entire community, with no exceptions, in all stages of the project, while paying attention to those whose voices are often marginalized, neglected, silenced, or overlooked.

Second, the children themselves approached the adults and the imams and asked them to provide a safe and secure play environment. As we hypothesized, this appeal aroused the adults’ emotions and motivated them to act (e.g., the response of the imams in Lakiya and their subsequent enlistment of the entire community). Third, the idea of setting up a playroom in the mosque was an unusual idea, which the adults we interviewed in the study (both professionals and women from the community) had never thought about on their own. This idea emerged from the children’s drawings. Thus, this positive deviance solution came from the most unexpected place. According to the PD approach, deviant ideas are found everywhere—among ordinary people and unusual suspects, not necessarily among professionals. In this project, for example, this original idea came from the children.

Moreover, including the children gave rise to the new and unique reframing of the topic of unintentional injuries by means of connecting two ostensibly different worlds of content: sacredness and play. The idea of setting up a playroom in a mosque, a religious and sacred site, is connected to the issue of preventing injuries, for not only is God sanctified in the mosque, but so are the lives of the children in the Bedouin community.

This study helped create, generate, and cascade a social network among three different Bedouin localities, thus creating ties between different communities in accordance with community-based participatory research. We chose to represent the social network disseminating these innovative practices from the inside out and from the bottom up, as is common in the PD approach [62, 63]. The change process is led by internal change agents who present their peers with “social proof” that problems can be solved. Given that the solutions emerge locally, they are more likely to be maintained and owned by potential adopters [64].

In the findings section, we chose to depict the social network in the form of a toy horse. In addition the reasons provided previously, as an entertainment-education artifact, the toy horse represents both play and movement, i.e., the diffusion of child safety ideas across the social networks of three Bedouin localities (Hura, Segev Shalom and Lakiya). Indeed, the idea of setting up a playroom in a mosque was born in Segev Shalom and Hura and implemented in Lakiya, illustrating this play and movement dimension of our study.

## Conclusions

This study reframed the official issue of physical safety into the notion of sacred and secure, enhanced the association between emotions and cognition by means of experiential approaches such as drama and entertainment, and stimulated creative thinking and the emergence of new and culturally relevant ideas and practices through positive deviance. Further, this study demonstrated the synergistic power of using a hybrid model that combined the rigor and vigor of three different health communication approaches to address a significant disparity in Israel with respect to the burden of childhood accidents. Our study generated solutions that emerged from and directly benefitted Bedouin children, who are at an overwhelmingly high risk of injury and death from preventable accidents.

### Limitations and future research

One of the limitations of this research may be that it was conducted only among three participating communities. Hence, any ideas and insights for dissemination need to be vetted and adapted by other Bedouin communities. Many of the PD solutions are contextual and hyper-local. The efficacy of these local practices (e.g., establishing a playroom in a mosque) in preventing injury must be assessed and monitored over time. Moreover, the hybrid model may not be readily accessible to all interventionists as they need to be familiar with all approaches and also must exhibit creativity in knowing which tool to use when and with which group. Such flexibility in culturally adapting research tools to the population is marked not just by creativity but also by patience in that bottom-up approaches require a great deal of time and investment in rapport-building.

## Supporting information

S1 Appendix The protocol for the community art-based focus group session. (PDF)

S2 Appendix Semi-structured protocol for professionals and other interested parties. (PDF)
