# Supplementary material for: Creating safe spaces to prevent unintentional childhood injuries among the Bedouins in southern Israel: A hybrid model comprising positive deviance, community-based participatory research, and entertainment-education
Source: PLoS One. 2021 Sep 22;16(9):e0257696. doi: 10.1371/journal.pone.0257696 (PMC8457499; doi:10.1371/journal.pone.0257696)
Supplement: S1 Appendix — (PDF) [file pone.0257696.s001.pdf]

S1 Appendix. The protocol for the community art-based focus group session

“Bottom to Top”: Using the **Positive Deviance** approach to identify positive behavioral practices in the Bedouin society in the Negev in order to promote a safer world for Bedouin children in Israel.

The research seeks to examine ideas and practices for improving childhood safety. The interview does not test knowledge and there are no right or wrong answers. Your participation in the research may significantly contribute to understanding the topic and developing a model which will help reduce children’s injuries in Bedouin society. All your identifying data will be confidential and will not be available to anyone apart from the research team. The interview will be recorded on a tape recorder and transcribed by research assistants to enable them to converse with you freely and focus on your words. The content will be used for research purposes only.

Exercise 1 – Warm-up game (with appropriate music playing in the background). Walk around the room, and when the music stops look for one person. State your name (if you don’t know the person), say <u>one special thing you do that is positive</u> and listen to what the other person standing in front of you says. When the music starts playing again, start walking and when it stops find someone else to meet.

Exercise time: five minutes.

Exercise 2 – Warm-up game: Who, like me, …?

Instruction: All participants sit on chairs that are arranged in a circle. There are no empty chairs. One participant stands at the center of the circle and asks a question beginning with the words “Who, like me, …?” (e.g., “Who, like me, likes to cook?”). Everyone who agrees with this statement must stand up and find somewhere else to sit. The standing participant who asked the question also finds somewhere to sit, so that another participant is left without a seat. The person without a seat now must stand at the center and ask the group a new question beginning with the words “Who is like me?”

Note 1: If no one stands up, the standing participant continues asking “Who, like me, …?” questions until other participants in the group have something in common.

Note 2: When participants stand up, they should not sit in the chairs that were next to their previous seat but rather look for a chair that is farther away.

Exercise time: 15 minutes.

Exercise 3: Sensations and emotions associated with the word “safety”

Instruction: All participants sit on chairs. The instructor asks each participant, in turn, to state an emotion or sensation related to the word “safety”.

The statue exercise: After the round eliciting associations with the word “safety”, the instructor asks each participant to stand up and perform a movement that expresses his or her sensation associated with the word “safety”. They do this one after the other, until a group statue is created.

The instructor photographs the group statue.

Exercise 4: The injury story: Creating a frozen image

The instructor asks a participant to tell about an accident in which a child was injured. If none of the participants has a story, the instructor tells a story about a child who was run over by a car that was backing up or a story about a child who drowned. The participants are then asked to demonstrate the situation by acting out the characters in the situation. To do so, the instructor repeats the story, and each actor mimics and performs the actions. The actors are then asked to freeze a frame of the injury story. The instructor circulates, touching the participants’ shoulder one by one and asking each to say how he or she feels.

Exercise 5: The power to change: The Joker (generative)

This time, the instructor asks each actor in turn to tell the story again, this time from his or her point of view. As each actor tells the story, anyone in the audience who wishes to change it can do so by shouting out the word “change” and then verbally suggesting a different solution. The actor then acts out the change or the person from the audience who made the suggestion switches places with the actor and performs the action differently.

Discussion: After all the participants have had a turn acting out the story, they discuss tips for change in the context of how “the story could have been told differently by finding solutions”.

Exercise 6 (or another session with the group): I would like to read you a letter written by a seven-year-old girl named Lynn:

“Hello, my name is Lynn. You don’t know me, but I know you. I know the important things you do in your work. I know there are many important things that need to be done in our society and many people talk about the subject of safety and don’t want us children to be hurt…. It’s important for me to tell you that sometimes we children spend many hours alone because our parents are busy doing housework or are at work. Because we are bored, we look for something to do…. You understand, we’re looking for something to do for ourselves…. But while we’re playing, we sometimes unintentionally do dangerous things and get hurt. What can you to provide us a safe environment where we can play?”

The instructor divides the group into pairs and asks each pair to propose three ideas for a safe play environment for children (outside as well as indoors, while using existing resources).

The group discusses the proposed ideas for finding a safe playing environment for the children.
