# Supplementary material for: Creating safe spaces to prevent unintentional childhood injuries among the Bedouins in southern Israel: A hybrid model comprising positive deviance, community-based participatory research, and entertainment-education
Source: PLoS One. 2021 Sep 22;16(9):e0257696. doi: 10.1371/journal.pone.0257696 (PMC8457499; doi:10.1371/journal.pone.0257696)
Supplement: S2 Appendix — (PDF) [file pone.0257696.s002.pdf]

S2 Appendix. Semi-structured protocol for professionals and other interested parties

"Bottom to Top": Using the **Positive Deviance** approach to identify positive behavioral practices in the Bedouin society in the Negev in order to promote a safer world for Bedouin children in Israel.

The research seeks to examine ideas and practices for improving children safety. The interview does not test knowledge and there are no right or wrong answers. Your participation in the research may significantly contribute to understanding the topic and developing a model which will help reduce children's injuries in the Bedouin society. All your identifying data will be confidential and will not be available to anyone apart from the research team. The interview will be recorded on a tape recorder and transcribed by research assistants to enable them converse with you freely and focus on your words. The content will be used for research purposes only.

<u>Warm-up questions:</u>

If you could make three wishes and have them come true in Bedouin society, what would you wish for?

<u>Attitudes regarding safety from childhood injury and intervention plans in Bedouin society:</u>

1) From the perspective of Bedouin society, what are the three main problems that must be handled?
2) If the interviewee did not mention the problem of children's injuries, ask: "You didn't mention the matter of children's injuries in Bedouin society. Why do you think this is not one of the three most critical problems from the perspective of Bedouin society?" If the interviewee does mention this problem, ask: "What do you think are the reasons and challenges that make it difficult for Bedouin society to create a safe environment for their children"?
3) What intervention plans were carried out or are currently being carried out in Bedouin society to prevent childhood injury?
4) Some say that the training provided by professionals or educational teams is somewhat useless because parents are familiar with the rules but find it difficult to follow them/ implement them. What do you think about this statement?
5) As far as you know, was an evaluation carried out to assess the effectiveness of training/ intervention plans? Are you aware of the findings of this evaluation? What were its recommendations?
6) In your opinion, are those providing safety training sufficiently familiar with the culture and nature of Bedouin society?

<u>Stories and projections:</u>

7) Can you please describe a case or cases of children's injury that affected you greatly?
8) When a child is injured, how do you think this affects the family? the close environment?
9) When such an incident happens, do the educational teams involved (kindergarten, school) discuss its social implications, or does the child receive mostly medical treatment?

10) Some claim that Bedouin parents are afraid of going to the hospital or reporting that their child was injured at home for fear of intervention by police or welfare authorities. Are you aware of such a claim? What do you think about it? Can you explain what the parents are afraid of?

I would like to read you a letter written by a seven-year-old girl named Lin:

“Hello, my name is Lynn. You don’t know me, but I know you. I know the important things you do in your work. I know there are many important things that need to be done in our society and many people talk about the subject of safety and don’t want us children to be hurt…. It’s important for me to tell you that sometimes we children spend many hours alone because our parents are busy doing housework or are at work. Because we are bored, we look for something to do…. You understand, we’re looking for something to do for ourselves…. But while we’re playing, we sometimes unintentionally do dangerous things and get hurt. What can you to provide us a safe environment where we can play?”

Please propose three ideas for a safe play environment for children (outside as well as indoors, while using existing resources)

<u>In the context of families</u>

11) Are you acquainted with a family whose child was injured and tell us about the difficult experience they went through?
12) Are you acquainted with a family that created a safe play area or safe environment for their child? This can also be the grandparents’ house.
